# WSPSorter: a real-time integrated system for non-destructive purity sorting of watermelon seeds based on rotary singulation and near infrared spectroscopy

**DOI:** 10.3389/fpls.2026.1909902

**Published:** 2026-08-19

**Authors:** Zhichao Ni, Jiakai Wan, Zebing Song, Yajie Liu, Shuangxu Yang, Bing Bai, Tingting Wu

**Affiliations:** 1 College of Mechanical and Electronic Engineering, Northwest A&F University, Yangling, China; 2 The Key Laboratory of Watermelon and Cabbage Digital Seed Industry, Ministry of Agriculture and Rural Affairs, P.R. China, Ningbo Weimeng Seed Industry Co., Ltd., Ningbo, China

**Keywords:** machine learning, near-infrared spectroscopy, non-destructive testing, seed sorting, watermelon seed purity

## Abstract

**Background:**

Watermelon seed purity is a critical determinant of varietal authenticity and production quality. In hybrid seed production, however, distinguishing between maternal seeds and hybrid seeds at the single-seed level remains challenging because their phenotypic differences are subtle. Conventional purity identification methods are often destructive, labor-intensive, and time-consuming and are poorly suited to the practical demand for rapid, continuous, single-seed online sorting.

**Methods:**

To address these challenges, this study developed the Watermelon Seed Purity Sorter (WSPSorter), an integrated real-time sorting system based on rotary singulation, near-infrared (NIR) spectroscopy, and pneumatic actuation. The system achieves stable single-seed delivery through a rotary seed-metering mechanism, acquires single-seed spectral information in real time, and combines NIR spectroscopy with an Extreme Gradient Boosting (XGBoost) classification model to discriminate maternal seeds from hybrid seeds.

**Results:**

The optimal model, combining wavelet denoising, the successive projections algorithm (SPA), and XGBoost, achieved accuracies of 98.89% on the validation set and 97.78% on the independent test set. WSPSorter achieved an average inter-sample interval of 254.88 ms, corresponding to a practical throughput of approximately 4.0 seeds per second. Sorting performance, evaluated against PCR-derived reference labels, reached 94.04% for maternal seeds and 90.05% for hybrid seeds.

**Discussion:**

These results indicate that WSPSorter effectively connects single-seed spectral sensing with real-time physical sorting, providing a feasible engineering solution for rapid, non-destructive, and automated purity sorting of watermelon seeds.

## 1 Introduction

Modern agricultural product processing increasingly requires online sorting systems that can identify individual materials without damage and immediately translate detection results into physical sorting actions. For seed processing, this requires the coordinated operation of stable single-seed delivery, rapid sensing, reliable classification, and accurately timed actuation; failure at any stage can reduce either sorting purity or practical throughput. This demand is particularly important in the watermelon seed industry. FAO statistics indicate that global watermelon production reached approximately 105 million tons in 2024 (Food and Agriculture Organization of the United Nations, 2024), while varietal purity directly affects genetic authenticity, population uniformity, field yield, and commercial quality. The Chinese national standard GB 16715.1–2010 therefore requires the varietal purity of watermelon parental seeds to be no less than 99.7% (Standardization Administration of China, 2010). Field identification and molecular marker methods provide valuable phenotypic and genetic verification (Lu et al., 2018), but they are primarily suited to batch-level quality assessment rather than rapid, continuous sorting of individual seeds. The key industrial challenge is thus not merely to determine seed purity, but to perform accurate, non-destructive, and real-time purity identification within a continuously operating sorting line.

Spectral sensing provides a feasible route for meeting these online detection requirements because it captures internal physicochemical information without damaging the seed (Hacisalihoglu and Armstrong, 2023). Combined with chemometrics or machine learning, spectral data have been used to associate seed characteristics with geographical origin, composition, vigor, and other quality traits (Zheng et al., 2026; Sousa et al., 2023; Qi et al., 2023). Applications now cover variety classification, grading, viability assessment, damage detection, and quality prediction in multiple crops (Wu et al., 2022; Silva et al., 2023; Wang and Song, 2023; Makmuang et al., 2024; Liu and Chen, 2026; Batista et al., 2022). HSI and NIR spectroscopy are the two principal approaches used for these tasks (Wang et al., 2021; Long et al., 2021). Although HSI can simultaneously provide spatial and spectral information for non-destructive seed assessment (Zhang et al., 2022), its high-dimensional data and computational burden complicate integration into high-throughput real-time sorting lines (Long et al., 2021). NIR spectroscopy, by contrast, offers rapid response, single-seed acquisition capability, sensitivity to absorption associated with chemical bonds such as C–H and N–H, and relatively straightforward integration with electromechanical equipment (Zhu et al., 2024). Nevertheless, much of the existing work emphasizes offline spectral discrimination, whereas industrial sorting additionally requires stable dynamic acquisition and precise synchronization between classification results and the corresponding moving material.

Watermelon seeds introduce additional material-specific difficulties to this online process. Their flat and irregular geometry increases the risk of overlap, multiple pickup, missed delivery, and unstable trajectories during continuous feeding. During dynamic spectral acquisition, changes in seed position and orientation can also affect illumination and diffuse-reflectance collection. At the same time, maternal seeds and hybrid seeds from the same variety exhibit only subtle external differences, making conventional appearancebased separation difficult. Previous optical studies on watermelon seeds have mainly addressed viability screening (Yasmin et al., 2019), bacterial infection (Lee et al., 2017), and quality grading (Liu et al., 2019), confirming that optical signals can capture relevant internal or external variation (Wang et al., 2021). However, these studies do not fully resolve the combined engineering problem of stable single-seed delivery, dynamically triggered spectral acquisition, purity discrimination, and physical sorting. Research directly associating individual-seed spectra with the purity status of phenotypically similar maternal and hybrid seeds remains limited (Long et al., 2021). Therefore, the unresolved gap concerns not only the classification model, but also the establishment of a complete real-time sensing-to-actuation chain adapted to watermelon seed characteristics.

To address this combined scientific and engineering gap, this study proposes a watermelon seed purity sorting method based on NIR spectroscopy. Our previous *Seedscreener* platform combined RGB imaging and NIR spectroscopy for automated morphological and biochemical phenotyping of individual wheat kernels, achieving a 94% acquisition success rate, prediction accuracies of 0.85 and 0.93 for biochemical and morphological traits, respectively, and a throughput of approximately 90 grains h^−1^ (Wu et al., 2023). Although that platform demonstrated the feasibility of coordinated single-kernel handling and multimodal sensing, it was designed for trait measurement rather than continuous purity sorting. The present study advances this foundation through three targeted developments. First, a rotary seed-metering mechanism and customized optical acquisition structure were designed for the flat and irregular geometry of watermelon seeds, enabling stable singulation and dynamically triggered NIR acquisition. Second, PCR-derived labels were linked to individual spectra to establish a WaveletSPA-XGBoost model for discriminating maternal seeds from hybrid seeds. Third, spectral acquisition, model inference, and pneumatic actuation were coordinated through event-synchronized control to create the Watermelon Seed Purity Sorter (WSPSorter). Scientifically, this work evaluates whether genotype-associated physicochemical differences can be captured indirectly through single-seed NIR spectra; technically, it establishes a continuous sensing–decision–actuation pipeline that converts spectral discrimination into real-time physical sorting.

## 2 Materials and methods

### 2.1 Plant materials and PCR-based purity identification

#### 2.1.1 Plant materials

The watermelon cultivar used in this study was ‘Meidu’ (Registration No. GPD Watermelon (2017) 330040), an F_1_ hybrid developed by crossing the female parent ZMF-20 with the male parent ZM-5. The experimental materials consisted of maternal seeds from the female parent and F_1_ hybrid seeds of ‘Meidu’. All seed materials were provided by Ningbo Weimeng Seed Co., Ltd. (Ningbo, Zhejiang, China) and were produced through artificial pollination in 2024.

#### 2.1.2 PCR amplification and purity labeling

Single-seed PCR was used to provide the reference labels for seed purity. Each seed was assigned a unique identifier during spectral acquisition to maintain a one-to-one correspondence between its spectrum and PCR result.

SSR primer pairs showing stable polymorphism between the maternal and hybrid seeds were selected as molecular markers (Lu et al., 2018).

For each preparation batch, the PCR premix was prepared by thoroughly mixing 800 *µ*L of 2× Taq Master Mix (Novoprotein, Suzhou, China), 800 *µ*L of ddH_2_O, and 12 *µ*L each of the forward and reverse primers (100 *µ*M). For each reaction, 9 *µ*L of the premix was aliquoted into a well, followed by the addition of 7 *µ*L of DNA template, yielding a final reaction volume of 16 *µ*L. The thermal cycling program consisted of an initial denaturation at 94°C for 3 min; 31 cycles of denaturation at 94°C for 30 s, annealing at 56°C for 15 s, and extension at 72°C for 30 s; and a final extension at 72°C for 5 min.

Amplification patterns were used to assign purity labels. Maternal seeds were defined as non-target seeds and assigned label 1, whereas F_1_ hybrid seeds were defined as target seeds and assigned label 0.

After matching spectra with PCR-derived labels, 1,200 valid samples were retained, including 600 maternal and 600 hybrid seeds. The dataset was divided into training, validation, and test sets at a ratio of 7:1.5:1.5, and final model performance was evaluated using the independent test set.

### 2.2 Hardware system design

**Figure 1:**
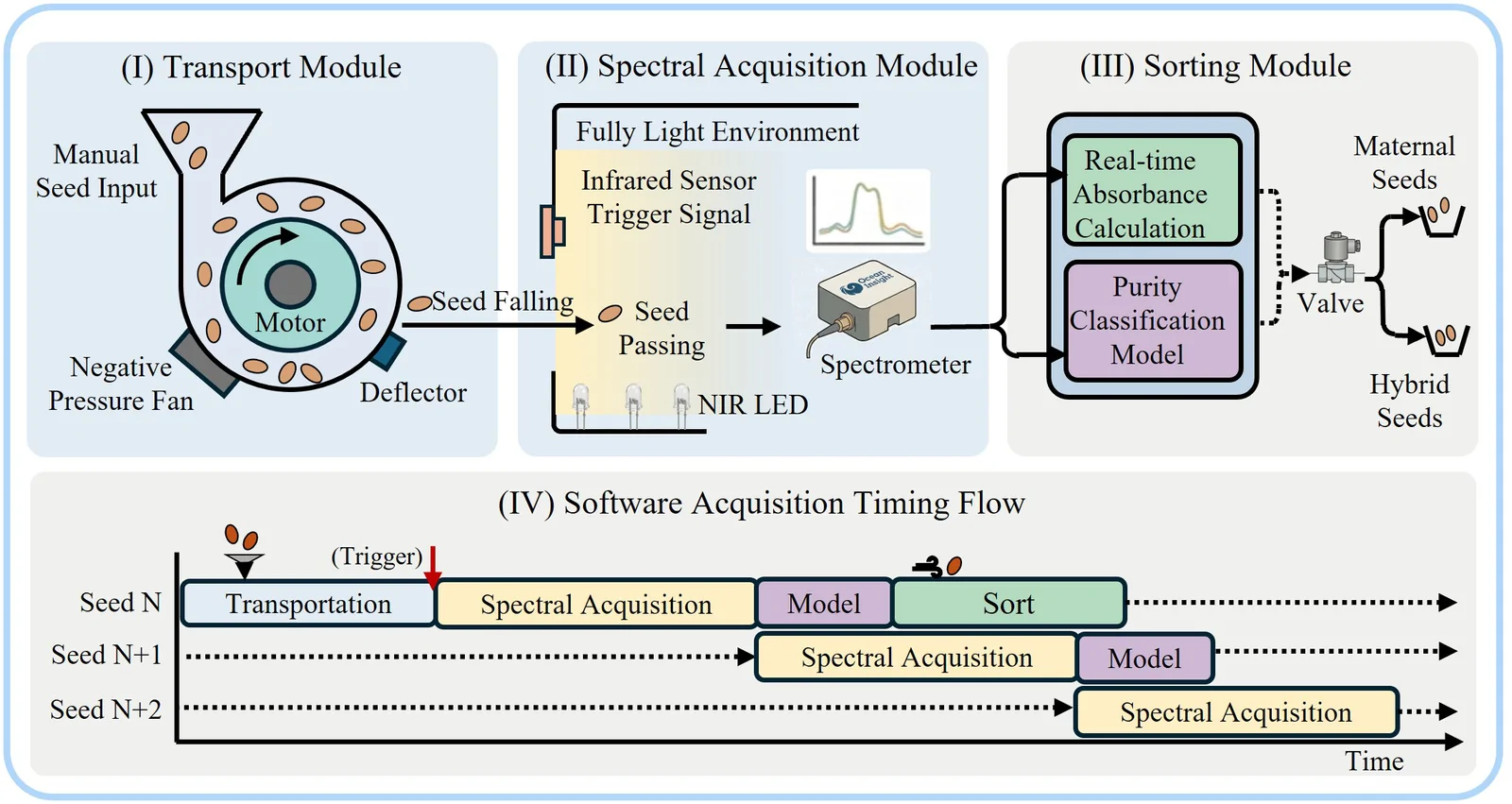
Basic operation flowchart of WSPSorter.

The automatic watermelon seed sorting system shown in **Figure 1** is designed for high-throughput processing of individual seeds. It consists of three hardware modules: a transportation module (I), a spectral acquisition module (II), and a sorting module (III). The overall workflow also includes a software acquisition timing flow (IV), which coordinates sensor triggering, spectral acquisition, model inference, and pneumatic actuation to synchronize continuous single-seed sorting.

The process initiates with watermelon seeds being fed into the funnel-shaped inlet in the transportation module (I), after which the negative-pressure fan and servo motor are turned on. Under negative pressure, each hole on the suction disc picks up one seed from the metering unit. The disc then rotates clockwise by approximately three-quarters of a revolution, carrying the seeds from the bottom of the container to the outlet. At the outlet, the seeds hit a deflector, leave the negative-pressure region, and fall into spectral acquisition module (II). Inside the enclosed illumination chamber, a pair of infrared sensors at the entrance detects each passing seed and sends a trigger signal to the spectrometer. The spectrometer then records the diffuse-reflectance spectrum of each seed in real time for subsequent purity classification.

After spectral acquisition, the seeds move into sorting module (III). The control software computes the absorbance of each seed in real time and generates a prediction using the purity classification model. The system then actuates a positive-pressure pneumatic device to direct the seeds into different channels according to the prediction result. The entire system operates as a continuous pipeline. While one seed is being measured, classified, and sorted, the next seed is simultaneously picked up and transported toward the spectral acquisition stage. With coordinated timing among trigger generation, spectral acquisition, model inference, and pneumatic ejection, the system enables continuous single-seed inspection and sorting.

#### 2.2.1 Seed transportation and singulation module

**Figure 2:**
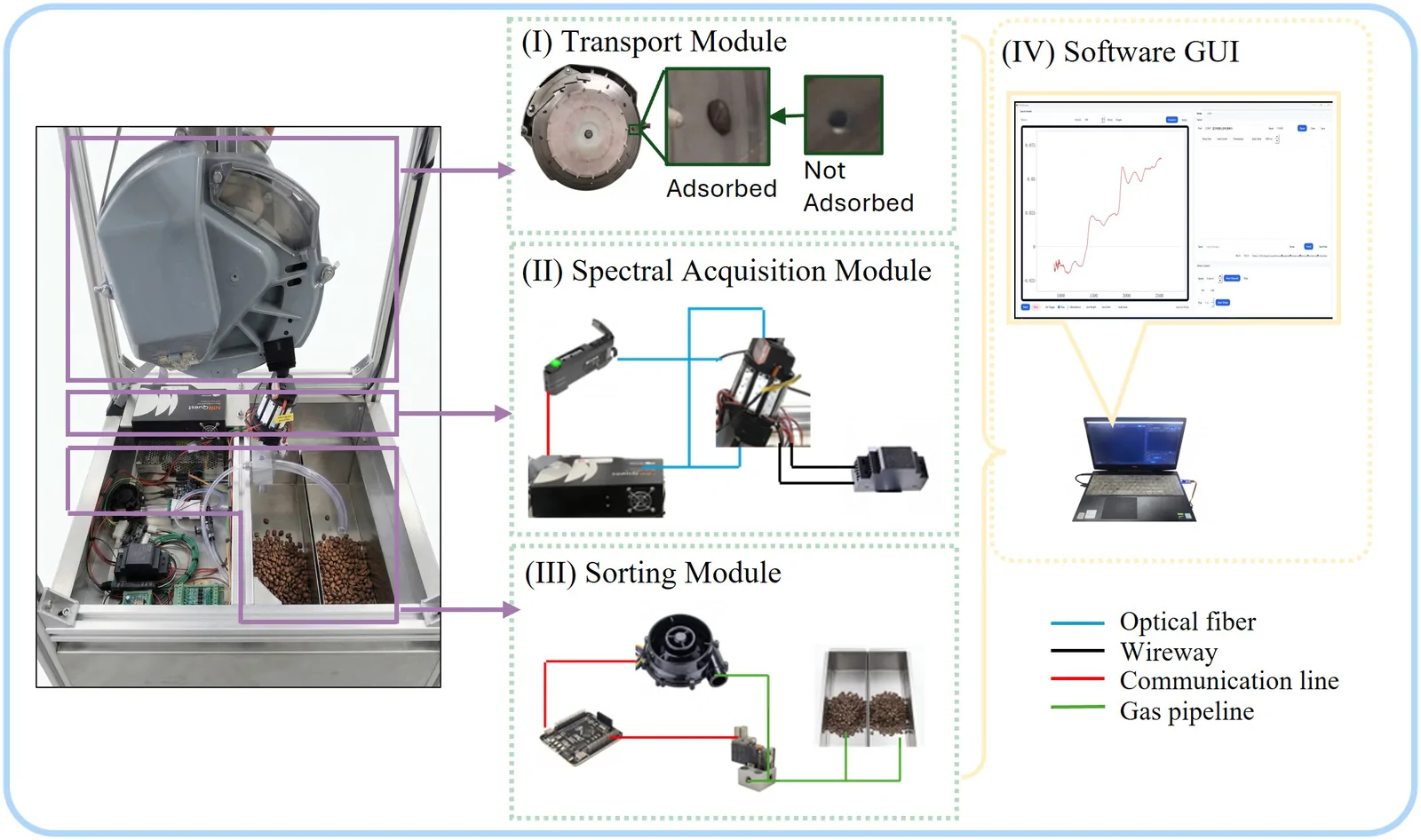
Physical prototype and connection schematic of WSPSorter.

Part I of **Figure 2** shows the transportation module, which consists of a circular suction plate, servo motor, negative-pressure fan, and protective housing. The 25-centimeter-diameter plate features 12 suction holes spaced at 30° intervals. Driven by the servo motor (up to 600 rpm) and a stable negative pressure of −4 kPa, the system reliably picks up and separates individual seeds from the inlet port, delivering them sequentially to the acquisition stage (Karayel et al., 2022; Raveendran et al., 2025).

#### 2.2.2 Spectral acquisition module

As illustrated in part II of **Figure 2**, the illumination system consists of an aluminum light pipe customized for watermelon seeds and equipped with 50 near-infrared LEDs that provide stable broadband illumination. Seeds fall through a 14 mm glass tube aligned with the center of the light pipe. Diffusereflectance signals are collected through a bifurcated Y-shaped optical fiber with a core diameter of 600 *µ*m and transmitted to a NIRQuest512 spectrometer (Ocean Optics, USA). The spectrometer covers a wavelength range of 900–2500 nm, provides 512 spectral channels, and has an approximate optical resolution of 5 nm. Although the instrument supports integration times ranging from 7 to 1500 ms, the integration time was fixed at 20 ms for all measurements. An infrared sensor positioned at the inlet detects each passing seed and triggers the spectrometer through an input/output (I/O) interface. Spectra were acquired under ambient laboratory conditions; neither temperature nor relative humidity was actively controlled during measurement.

#### 2.2.3 Pneumatic sorting and execution module

Once spectral data are acquired for a seed, the sorting module begins real-time analysis. The classification model preprocesses the raw spectrum and extracts features to assess purity, and the prediction then determines the sorting action. As shown in part III of **Figure 2**, if a seed is identified as a maternal seed (Label 1), the actuator remains still. The seed follows its natural trajectory and falls into the collection box directly below. For a seed identified as a target hybrid seed (Label 0), the system triggers a solenoid valve during a specific timing window. The resulting air jet deflects the seed into a collection bin located diagonally below, completing the sorting process.

### 2.3 Integrated software system development

**Figure 3:**
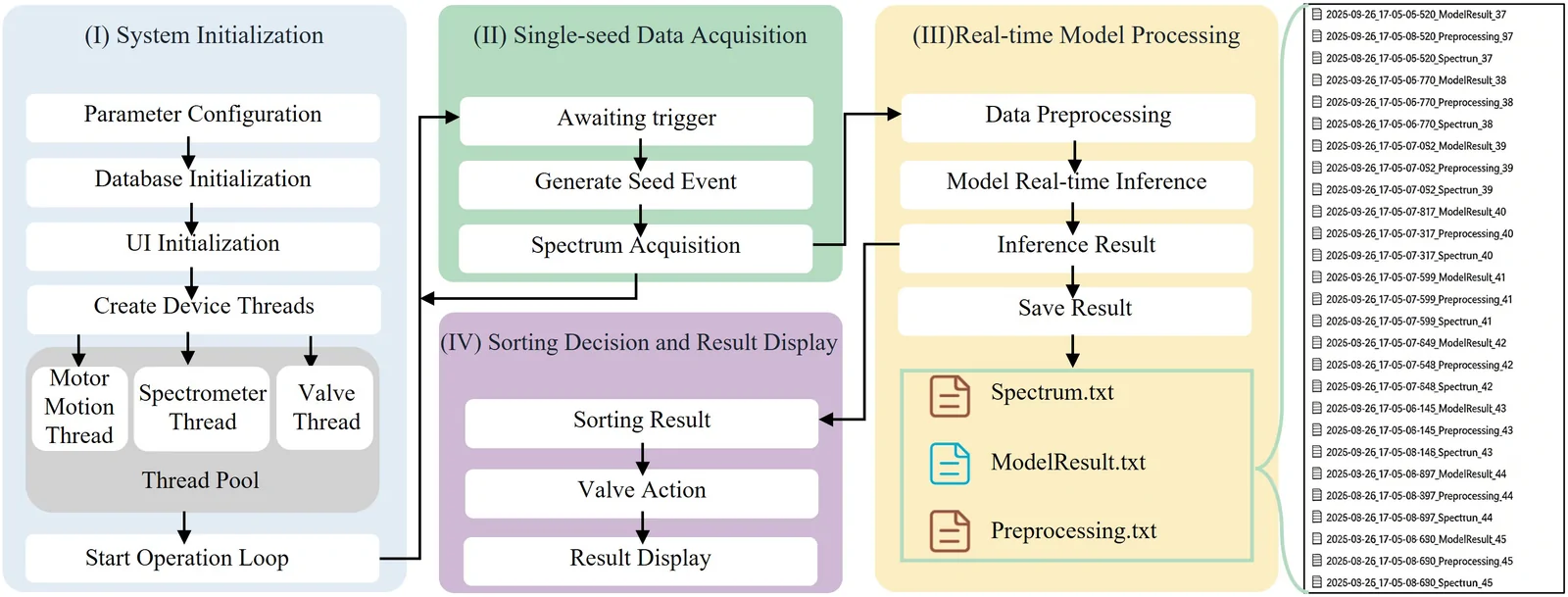
Software architecture of WSPSorter.

To provide closed-loop control for seed sorting, we developed an integrated software system structured around four primary modules, as illustrated in **Figure 3**. The workflow begins with system initialization (I), which executes parameter configuration for rapid sorting adjustments, followed by database and user interface (UI) initialization. The system then creates dedicated device threads for motor motion, the spectrometer, and the valve, managing them within a thread pool before starting the continuous operation loop. Within this loop, the single-seed data acquisition module (II) awaits a sensor trigger to generate a seed event and execute spectral acquisition. The data are subsequently routed to the real-time model processing module (III) for preprocessing and model inference, with the inference results and spectral data continuously saved to designated text files (Spectrum.txt, ModelResult.txt, and Preprocessing.txt). Finally, the sorting decision and result display module (IV) translates the inference result into a sorting command that drives the valve. The system updates the UI with the final result and then returns to await the next trigger, ensuring continuous and robust operation.

### 2.4 Spectral processing and purity modeling

Near-infrared (NIR) spectroscopy is commonly used to establish correlations with seed components such as protein, fat, and moisture (Yang et al., 2024a; Hacisalihoglu and Armstrong, 2023). Because these physicochemical traits and their associated spectral responses can vary with genetic background (Yang et al., 2024b), seeds from different parental backgrounds may exhibit subtle but characteristic physicochemical differences. The present study therefore constructs a direct “spectrum–purity” bridge for a commercial hybrid watermelon variety. Using maternal and hybrid seeds as two genetic sources, a binary purity classification model was established without requiring separate quantitative analyses of individual physicochemical components. This study thereby evaluates the feasibility of rapid single-seed purity identification using NIR spectroscopy.

In the experiment, absorbance spectra were acquired from 1,200 watermelon seeds (600 maternal seeds and 600 hybrid seeds) using the equipment developed in this study. The samples were partitioned into training, validation, and test sets in a 7:1.5:1.5 ratio. During the training phase, the optimal model hyperparameters were determined via cross-validation. Spectral preprocessing included spectral calibration, absorbance conversion, noise elimination, and feature extraction. Specifically, the raw lightintensity signals were first calibrated using Equation 1 to obtain reflectance, which was then converted into absorbance spectra using Equation 2.

(1) $$R(\lambda )=\frac{I_{s}(\lambda )-I_{d}(\lambda )}{I_{l}(\lambda )-I_{d}(\lambda )}$$

(2) $$A(\lambda )=\underset{10}{\log}(\frac{1}{R(\lambda )})$$

where *A* is the absorbance spectrum, *R* is the diffuse reflectance, *I_s_* is the reflected light intensity, *I_d_* is the dark reference intensity, and *I_l_* is the light reference intensity. To reduce random noise and improve model stability, discrete wavelet threshold denoising was applied to the absorbance spectra (Donoho, 1995; Xia et al., 2026). Specifically, each spectrum was subjected to a three-level discrete wavelet decomposition, and soft-thresholding was applied to the detail coefficients at each level using the universal threshold. The universal threshold and the corresponding soft-thresholding function are given in Equation 3. The denoised spectrum was then reconstructed by inverse discrete wavelet transform. The thresholding procedure is defined as follows:

(3) $$\lambda =\sigma \sqrt{2\text{ln }N},\;\hat{w}=\{\begin{array}{ll} \text{sgn}(w)(|w|-\lambda ), & |w|\geq \lambda ,\\ 0, & |w|<\lambda , \end{array}$$

where *λ* is the universal threshold, *N* is the spectral length, *w* is the original detail coefficient, *w* is b the thresholded coefficient after soft-thresholding, and sgn(·) denotes the sign function. In this study, *σ* represents the estimated noise standard deviation. After thresholding, the denoised absorbance spectrum was reconstructed from the retained approximation coefficients and the thresholded detail coefficients.

To improve model training efficiency and reduce collinearity in the spectral data, the Successive Projections Algorithm (SPA) was employed for feature selection (Araújo et al., 2001). The candidate wavelength set comprised all sampling points within eight spectral ranges: 1160–1180, 1200–1220, 1440– 1460, 1500–1530, 1720–1760, 1930–1950, 2050–2180, and approximately 2300–2350 nm. SPA selects variables from these ranges in a forward iterative manner by projecting each candidate wavelength onto the orthogonal complement of the subspace spanned by the already selected variables. At each iteration, the wavelength with the largest projection residual is retained, thereby reducing redundancy among variables while preserving informative spectral features. The core projection procedure is given in Equation 4:

(4) $$\begin{array}{ll} P & =X_{s}{\left(X_{s}^{T}X_{s}\right)}^{-1}X_{s}^{T},\\ E & =I-P,\\ r_{j} & =∥Ex_{j}{∥}_{2},\\ j_{\text{next}} & =\text{arg }\underset{j\in C}{\max}\{r_{j}\}, \end{array}$$

where *X_s_* is the matrix composed of the previously selected wavelength vectors; *P* is the projection matrix onto the column space of *X_s_*; *I* is the identity matrix; *E* is the projection matrix onto the orthogonal complement of the column space of *X_s_*; *x_j_* is the column vector corresponding to the *j*-th candidate wavelength; *r_j_* is the Euclidean norm of the residual vector of *x_j_* after projection; *C* denotes the index set of the remaining candidate wavelengths; and *j*_next_ is the index of the wavelength selected in the current iteration. Candidate subsets containing different numbers of wavelengths were compared by crossvalidation. The final subset of 26 discrete wavelengths was retained because it provided the best balance between classification performance and feature dimensionality while minimizing collinearity through the maximum-projection-residual criterion.

Seed purity is treated discrimination as a binary classification task, with single-seed PCR results serving as ground-truth labels. Label 1 denotes non-target maternal seeds, whereas Label 0 denotes target hybrid seeds. The classification model uses preprocessed absorbance spectra to output a binary label.

A machine learning classification model was then used to predict seed purity. Considering the complex nonlinear relationships typically present between near-infrared spectral features and seed genetic phenotypes, the Extreme Gradient Boosting (XGBoost) algorithm was selected to construct the binary classification model (Chen and Guestrin, 2016). Within the gradient-boosting framework, the algorithm sequentially generates decision trees that fit the residuals from preceding iterations. The inclusion of a regularization term in the objective function controls model complexity, promotes rapid convergence, and reduces the risk of overfitting. In this study, the preprocessed data are used to train the XGBoost classifier through supervised learning, establishing a nonlinear mapping between spectral features and seed purity and ultimately producing a binary classification result for each watermelon seed.

The optimized hyperparameters of the classification models used for performance comparison are summarized in **Table 1**. These values were selected through cross-validation, and hyperparameters not listed in the table were retained at their default values.

**Table 1:** Optimized hyperparameters of the classification models.

Model	Optimized hyperparameters
SVM	C = 10.0; gamma = 0.01
LR	C = 0.1
KNN	N_neighbors = 5; weights = distance
XGBoost	n_estimators = 200; learning_rate = 0.05; max_depth = 3; subsample = 0.8; colsample_bytree = 0.8

### 2.5 Evaluation metrics for classification models

To evaluate the classification performance of the watermelon seed purity discrimination model, accuracy, precision, F1-score, and Cohen’s kappa coefficient were adopted as the primary metrics. Accuracy reflects overall classification correctness, precision measures the purity of seeds predicted as the target hybrid-seed class, and the F1-score evaluates the balance between precision and recall. Cohen’s kappa measures the agreement between model predictions and PCR-derived reference labels after correcting for agreement expected by chance. In this study, target hybrid seeds to be retained were defined as the positive class, whereas maternal seeds to be rejected were defined as the negative class. The confusion-matrix terms are true positive (TP), true negative (TN), false positive (FP), and false negative (FN). Because the primary objective of the sorting task is to minimize impurity among the retained target seeds, precision was considered particularly important. The evaluation metrics are defined as follows (Equations 5–8):

(5) $$\text{Accuracy}=\frac{TP+TN}{TP+TN+FP+FN}$$

(6) $$\text{Precision}=\frac{TP}{TP+FP}$$

(7) $$F_{1}=\frac{2TP}{2TP+FP+FN}$$

(8) $$\kappa =\frac{2(TP\times TN-FP\times FN)}{\left(TP+FP)(FP+TN)+(TP+FN)(FN+TN\right)}$$

Here, TP denotes target seeds correctly classified as positive, TN denotes non-target seeds correctly classified as negative, FP denotes non-target seeds incorrectly classified as positive, and FN denotes target seeds incorrectly classified as negative.

## 3 Results

### 3.1 Single-seed delivery and spectral acquisition stability

Stable real-time purity sorting relied on continuous single-seed delivery and reliable spectral acquisition. To verify the continuous release stability of the seed discharger, ten groups of repetitive experiments were conducted using 100 watermelon seeds per group. Results indicated that all seeds were completely discharged without simultaneous release of two seeds or obvious missed releases, demonstrating the mechanism’s stable and continuous single-seed release capability. This performance provided stable seed supply conditions for subsequent real-time detection.

**Figure 4:**
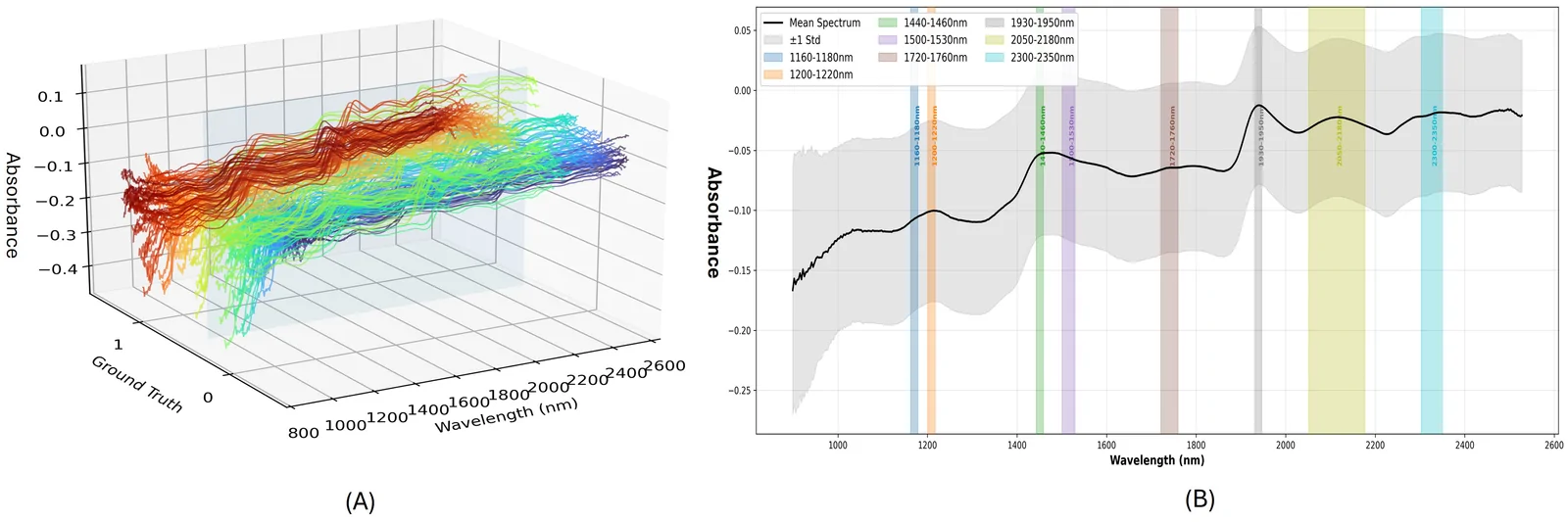
Spectral results. **(A)** Original spectral data, **(B)** original spectral data with selected characteristic wavelengths.

During the batch sampling process, WSPSorter successfully completed real-time spectral acquisition for all tested watermelon seeds, with no sample omission or acquisition failure observed, confirming stable and precise coordination among the seed discharge, trigger control and spectral acquisition modules to enable reliable single-seed detection. As shown in **Figure 4A**, the raw spectra of different seed samples exhibited consistent overall trends but notable variations in specific local bands, demonstrating that near-infrared spectroscopy can effectively characterize the purity-related information of watermelon seeds. Wavelet denoising was applied to reduce random spectral fluctuations while preserving the main absorbance features. SPA was then applied to the candidate sampling points within the eight specified spectral ranges and retained 26 discrete characteristic wavelengths based on the maximum-projectionresidual criterion and cross-validation performance, as shown in **Figure 4B**. The selected wavelengths provided a compact and minimally collinear feature set for subsequent purity classification.

The selected spectral regions can be tentatively associated with overtone or combination absorptions of hydrogen-containing groups (Osborne et al., 1993). Representative wavelengths near 1168.723, 1210.795, 1740.441, and 2299.643 nm are mainly related to C–H vibrations associated with lipids; wavelengths near 1449.330 and 1940.543 nm are associated with O–H absorption related to moisture; and wavelengths near 1513.521 and 2051.202 nm are associated with N–H absorption related to proteins. Lipid-associated regions account for the largest proportion of the selected wavelengths, which is consistent with the oil-rich characteristics of watermelon seeds. Differences in genetic background between maternal selfed seeds and filial hybrid seeds may affect lipid synthesis and related metabolic processes, thereby producing physicochemical differences that can be indirectly captured by NIR spectra (Yang et al., 2024b). Because these components were not independently quantified, the assignments should be interpreted as spectroscopic associations rather than direct compositional confirmation.

**Figure 5:**
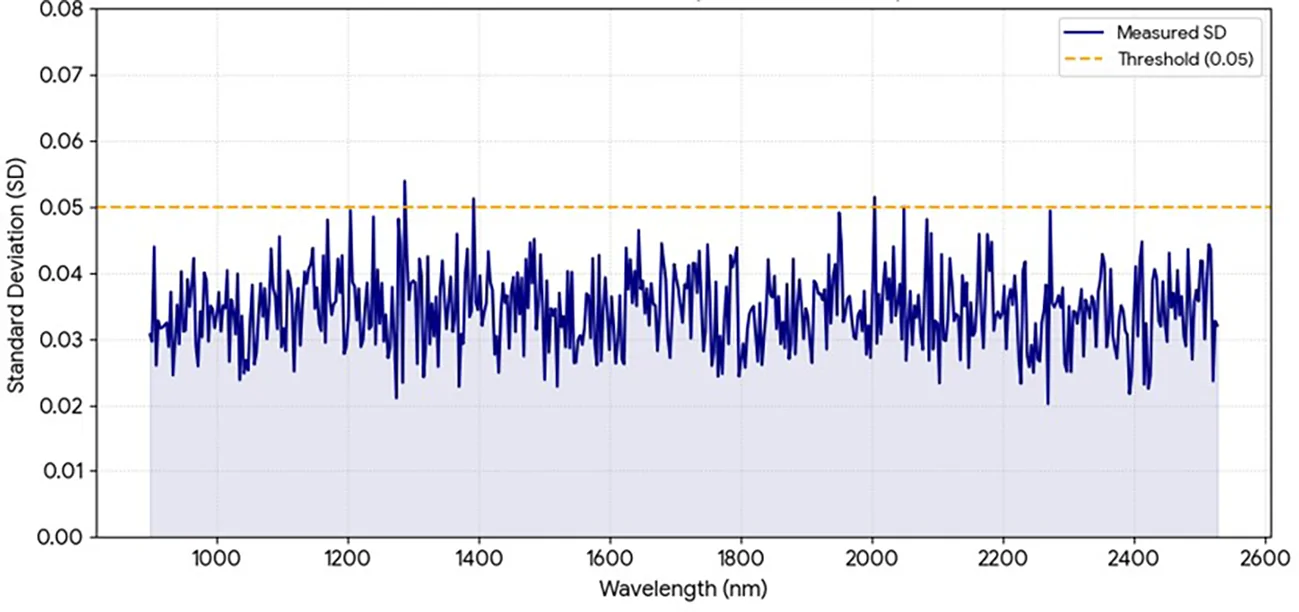
Standard deviation of repeated single-seed spectral measurements.

To further evaluate sampling consistency, a single seed was selected for 20 consecutive measurements, as illustrated in **Figure 5**. The results show that the standard deviation across all bands is generally below 0.05, confirming that the system possesses high repeated sampling stability.

### 3.2 Purity classification model construction and performance evaluation

After stable single-seed spectral data were acquired, watermelon seed purity classification models were constructed and evaluated. Because the selection of preprocessing methods and classification algorithms substantially influences model performance, multiple combinations were compared. The evaluated classifiers were support vector machine (SVM), logistic regression (LR), k-nearest neighbors (KNN), and XGBoost. The preprocessing methods included no preprocessing, Savitzky–Golay smoothing (SG), wavelet denoising, and Z-score standardization. The reported metrics comprise validation accuracy (Val Acc), test accuracy (Te Acc), test precision (Te Prec), test F1-score (Te F1), and Cohen’s kappa coefficient. As shown in **Table 2**, the XGBoost model with wavelet preprocessing and SPA-selected features achieved the best overall performance, with a validation accuracy of 0.9889, a test accuracy of 0.9778, a precision of 0.9886, an F1-score of 0.9775, and a kappa coefficient of 0.9556. These results demonstrate both strong classification performance and high agreement with the PCR-derived reference labels beyond chance.

**Table 2:** Performance comparison of classification models with SPA-selected features.

Preproc	Model	Confusion matrix (test)				Performance metrics				
		TP	TN	FP	FN	Val Acc	Te Acc	Te Prec	Te F1	Kappa
NONE	SVM	80	62	28	10	0.8556	0.7889	0.7407	0.8081	0.5778
	LR	59	70	20	31	0.7778	0.7167	0.7468	0.6982	0.4333
	KNN	73	75	15	17	0.9222	0.8222	0.8295	0.8202	0.6444
	XGBoost	84	88	2	6	0.9611	0.9556 0.9556	0.9767	0.9545	0.9111
SG	SVM	85	60	30	5	0.8556	0.8056	0.7391	0.8293	0.6111
	LR	52	77	13	38	0.7722	0.7167	0.8000	0.6710	0.4333
	KNN	74	81	9	16	0.9222	0.8611	0.8916	0.8555	0.7222
	XGBoost	84	85	5	6	0.9611	0.9389	0.9438	0.9385	0.8778
WAVELET	SVM	88	60	30	2	0.8556	0.8222	0.7458	0.8462	0.6444
	LR	52	81	9	38	0.7722	0.7389	0.8525	0.6887	0.4778
	KNN	75	82	8	15	0.9222	0.8722	0.9036	0.8671	0.7444
	XGBoost	87	89	1	3	**0.9889**	**0.9778**	**0.9886**	**0.9775**	**0.9556**
Z-SCORE	SVM	83	60	30	7	0.8556	0.7944	0.7345	0.8177	0.5889
	LR	53	80	10	37	0.7778	0.7389	0.8413	0.6928 0.6928	0.4778 0.4778
	KNN	75	82	8	15	0.9222	0.8722	0.9036	0.8671 0.8671	0.7444 0.7444
	XGBoost	70	84	6	20	0.8333	0.8556 0.8556	0.9211	0.8434 0.8434	0.7111 0.7111

Bold values indicate the best performance for each metric across all preprocessing methods and classification models.

Analysis of the feature selection results showed that the 26 characteristic wavelengths were not uniformly distributed across the full spectrum, but were concentrated in several local absorbance intervals. This distribution suggests that purity-related spectral information is mainly associated with specific wavelength regions rather than the entire spectral range. The results further indicate that wavelet denoising and the successive projections algorithm (SPA) effectively reduced spectral redundancy while preserving the most informative variables for purity discrimination.

Based on the combined performance on the validation and test sets, Wavelet-SPA-XGBoost was identified as the optimal purity classification model. This result demonstrates that single-seed near-infrared spectroscopy provides an effective means of distinguishing watermelon seed purity. Consequently, this model was integrated into the WSPSorter online control system for subsequent real-time discrimination and sorting control.

The real-time processing procedure for single-seed purity discrimination and sorting is summarized in **Algorithm 1**. During real-time deployment, the classifier retained the preprocessing and feature-selection workflow used in training and directly output a binary label for each seed, where label 0 denotes a hybrid seed and label 1 denotes a maternal seed.

Algorithm 1

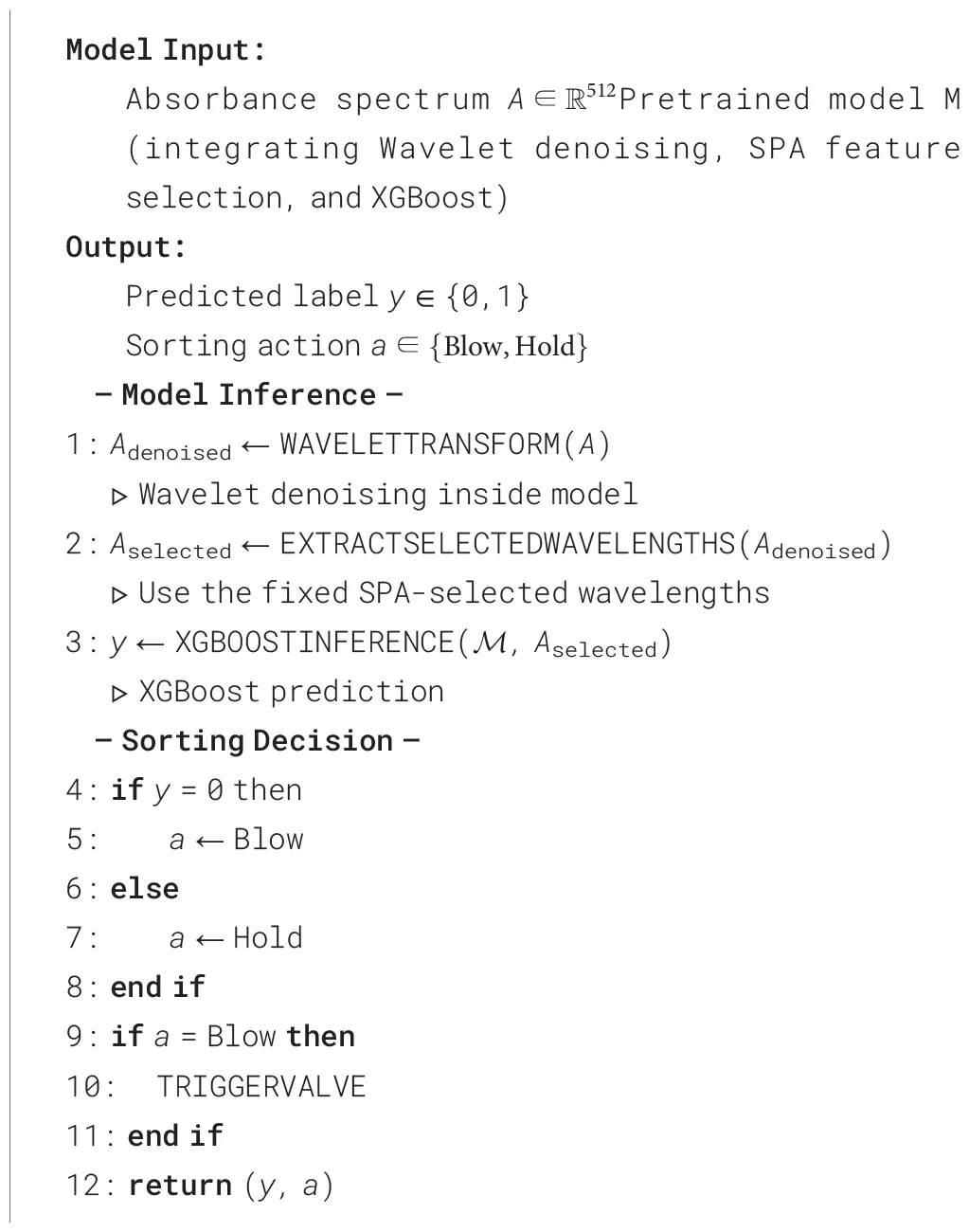

### 3.3 Real-time throughput and sorting performance

The real-time performance of WSPSorter relies on the coordinated execution of seed transport, spectral acquisition, model inference, and pneumatic actuation. For each seed, the sorting process includes two successive motion stages: passage through the light-source channel (180 ms) and post-exit travel to the sorting outlet (120 ms). Spectral acquisition is completed during passage through the light-source channel and is therefore included within the 180 ms transit period, whereas model inference and solenoid-valve response are carried out in parallel during the subsequent 120 ms post-exit stage. Accordingly, the ideal total processing time for a single seed is 300 ms. As illustrated in section IV of **Figure 1**, WSPSorter operates in a continuous pipelined mode, in which acquisition of the next seed begins immediately after completion of the current sampling event, rather than waiting for the entire sorting cycle to finish.

As presented in **Table 3**, a throughput evaluation was conducted on the developed system by continuously executing 1,000 acquisition cycles, with the exact timestamps recorded for statistical analysis. Based on the elapsed time between the first and the last acquisition timestamps, the average interval between two adjacent acquisition events was 254.88 ms, corresponding to an operational throughput of approximately 4.0 seeds *s*^−1^.

**Table 3:** Summary of throughput-related timing parameters of WSPSorter. Measured values are reported as mean ± standard deviation.

Item	Value	Unit/description
Number of acquisition cycles	1000	cycles
Average model inference time	0.011 ± 0.002	ms
Average interval between adjacent acquisition events	254.88 ± 12.16	ms
Operational throughput	3.92 ± 0.20	seeds *s*^−1^

We evaluated the real-time sorting performance of WSPSorter under practical operating conditions using pure samples of maternal seeds and hybrid seeds. As shown in **Table 4**, the system processed 436 maternal seeds and acquired 436 spectral datasets. During the maternal-selfed-seed test, the solenoid valve was triggered 27 times, 26 seeds were blown into the target box, and 410 seeds were retained in the nontarget box, corresponding to a decision accuracy of 93.81% and a final sorting accuracy of 94.04%. In the hybrid-seed test, 583 spectral datasets were obtained from 583 seeds. The solenoid valve was triggered 531 times, with 525 seeds entering the target box and 58 reaching the non-target box. This corresponds to a decision accuracy of 91.08% and an end-to-end sorting accuracy of 90.05%. These results indicate that total system errors stem not only from model discrimination but also from mechanical execution errors, such as trajectory deviations after blowing or collisions at the outlet.

**Table 4:** Results of real-time sorting under pure-sample tests.

Sample type	Input	Spectra	Valve	Target box	Non-target box	Decision acc. (%)	Sorting acc. (%)
Maternal selfed seeds (Non-target)	436	436	27	26	410	93.81	94.04
Hybrid seeds (Target)	583	583	531	525	58	91.08	90.05

## 4 Discussion

### 4.1 Engineering significance and system feasibility of WSPSorter

The principal engineering contribution of WSPSorter lies in demonstrating the feasibility of closing the loop between single-seed spectral sensing and real-time physical sorting. By integrating seed delivery, triggered spectral acquisition, model inference, and pneumatic actuation into one platform, WSPSorter establishes a complete perception-to-execution pipeline for non-destructive purity screening (Wu et al., 2023). The stable sampling performance observed across watermelon seeds, with a standard deviation below 0.05, indicates that the system can provide sufficiently consistent inputs for downstream decisionmaking. In addition, the achieved throughput of 4.0 seeds *s*^−1^ confirms that single-seed near-infrared sensing can be synchronized with online control under continuous operation (Yasmin et al., 2022).

While the current throughput remains a bottleneck compared to large-scale commercial machinery, WSPSorter stands as a critical proof-of-concept milestone. Conventional purity assessments rely on molecular methods such as SSR or PCR, which, despite their accuracy, are fundamentally destructive, labor-intensive, and time-consuming (Wang et al., 2021; Kamaşak et al., 2022). By enabling automated, single-seed prescreening prior to molecular confirmation, WSPSorter serves as an efficient upstream quality-control tool that significantly reduces the burden of destructive testing (Long et al., 2021). Most importantly, it successfully bridges the gap between spectral analysis and real-time execution, representing a crucial step in transitioning non-destructive single-seed sorting from laboratory research to industrial application (Yasmin et al., 2022).

### 4.2 Interpretation of the spectral classification results

The classification results indicate that single-seed near-infrared spectra contain stable and exploitable information for purity discrimination (Zhang et al., 2022; Reddy et al., 2023; Matsuda and Ohara, 2023; Zhu et al., 2024). Among all tested combinations, the XGBoost model based on wavelet-preprocessed spectra and SPA-selected variables achieved the best overall performance, with high classification accuracy and high precision for the target hybrid-seed class. Its F1-score of 0.9775 confirms a favorable balance between precision and recall, while the kappa coefficient of 0.9556 indicates high agreement between model predictions and PCR-derived labels beyond chance. This indicates that the superiority of the final model arises not only from the classifier itself, but from the combined effect of noise suppression, variable selection, and nonlinear discrimination. Wavelet preprocessing likely improves discrimination by suppressing spectral noise and enhancing local absorbance differences, whereas SPA reduces redundancy and retains the most informative wavelengths (Yan, 2025; Xia et al., 2026; Zheng et al., 2026). On this basis, XGBoost can more effectively model the nonlinear relationships between characteristic spectral variables and purity labels.

At the same time, the biological meaning of this classification should be interpreted carefully. Nearinfrared spectroscopy does not directly measure genotype; rather, it captures chemical and physical traits that are associated with the underlying genetic background (Sousa et al., 2023; Hacisalihoglu and Armstrong, 2023; Yang et al., 2024b). In other words, the proposed model identifies purity-related phenotypic expressions instead of performing direct molecular verification. This distinction is important because it defines both the strength and the boundary of the method. The strength lies in enabling rapid and non-destructive real-time discrimination, whereas the boundary is that reliable PCR-based labeling remains necessary for model training and periodic validation (Kamaşak et al., 2022). Accordingly, nearinfrared sensing should be considered a surrogate but operationally efficient approach for real-time batch inspection, rather than a substitute for molecular identification.

### 4.3 Practical implications, current limitations, and future directions

The sorting experiments further show that the current bottleneck of WSPSorter is no longer mainly the classification model, but the physical execution stage of the integrated system. In the pure-sample tests, almost 94% maternal seeds were correctly routed, whereas hybrid seeds achieved high algorithmic decision accuracy but slightly lower end-to-end sorting accuracy. This gap indicates that part of the residual error originates after classification, during seed ejection and physical routing. The most likely causes are post-ejection trajectory drift, outlet collision, and imperfect synchronization between seed motion and pneumatic actuation (Mukasa et al., 2024; Faqeerzada et al., 2020; Chen et al., 2024). Therefore, additional gains in practical sorting performance are more likely to come from mechanical and control optimization than from further minor adjustments to the classifier itself.

A second limitation concerns data scope and model generalizability. Because PCR labeling is expensive and labor-intensive, the present model was developed and evaluated using seeds from a single watermelon variety and should therefore be regarded as variety-specific. Direct application to other varieties may reduce detection accuracy because differences in genetic background, seed-coat structure, moisture, and chemical composition can shift spectral distributions and alter the effectiveness of the selected wavelengths (Tu et al., 2022; Yang et al., 2024b). Future work should therefore focus on two complementary directions. First, the mechanical subsystem should be refined by optimizing outlet geometry, ejection trajectory stability, and actuation timing. Second, multi-variety and multi-batch datasets should be established for external validation. A small number of PCR-labeled samples from each target variety may then be used to recalibrate the model, re-evaluate the SPA-selected wavelengths, and reduce inter-variety spectral differences through spectral standardization or feature alignment.

## 5 Conclusion

WSPSorter integrates rotary single-seed delivery, triggered NIR acquisition, Wavelet-SPA-XGBoost classification, and pneumatic actuation into a unified real-time purity-sorting system. Based on 1,200 PCR-labeled watermelon seeds from a single variety, the optimal model achieved a validation accuracy of 98.89%, a test accuracy of 97.78%, a target-class precision of 98.86%, an F1-score of 97.75%, and a kappa coefficient of 0.9556. During continuous operation, the system achieved an average inter-sample interval of 254.88 ms and a practical throughput of approximately 4.0 seeds *s*^−1^. End-to-end sorting accuracies reached 94.04% for maternal seeds and 90.05% for hybrid seeds. These findings demonstrate that genotype-associated physicochemical differences can be captured indirectly through single-seed NIR spectra and translated into real-time physical sorting decisions. The principal contribution of this study is therefore not only the development of an accurate spectral classifier, but also the establishment of a complete sensing-to-actuation pipeline for non-destructive seed purity screening. Although multi-variety validation and model adaptation are still required, WSPSorter provides a practical foundation for reducing dependence on destructive molecular testing and advancing intelligent quality control in the seed industry.

## Data Availability

The raw data supporting the conclusions of this article will be made available by the authors, without undue reservation.
